# Improving Skin Cancer Diagnostics Through a Mobile App With a Large Interactive Image Repository: Randomized Controlled Trial

**DOI:** 10.2196/48357

**Published:** 2023-08-09

**Authors:** Gustav Gede Nervil, Niels Kvorning Ternov, Tine Vestergaard, Henrik Sølvsten, Annette Hougaard Chakera, Martin Grønnebæk Tolsgaard, Lisbet Rosenkrantz Hölmich

**Affiliations:** 1 Department of Plastic Surgery Herlev-Gentofte Hospital Herlev Denmark; 2 Department of Dermatology and Allergy Centre Odense University Hospital Odense Denmark; 3 Dermatology Centre North Aalborg Denmark; 4 AGATA Private Hospital Copenhagen Denmark; 5 Copenhagen Academy for Medical Education and Simulation Copenhagen University Hospital Rigshospitalet Copenhagen Denmark; 6 Department of Obstetrics Copenhagen University Hospital Rigshospitalet Copenhagen Denmark; 7 Department of Clinical Medicine University of Copenhagen Copenhagen Denmark

**Keywords:** dermoscopy, nevi, skin neoplasms, benign skin tumors, melanoma, skin cancer, medical education, eLearning, digital learning, diagnostic test, mHealth, mobile app, recognition training, skin lesions

## Abstract

**Background:**

Skin cancer diagnostics is challenging, and mastery requires extended periods of dedicated practice.

**Objective:**

The aim of the study was to determine if self-paced pattern recognition training in skin cancer diagnostics with clinical and dermoscopic images of skin lesions using a large-scale interactive image repository (LIIR) with patient cases improves primary care physicians’ (PCPs’) diagnostic skills and confidence.

**Methods:**

A total of 115 PCPs were randomized (allocation ratio 3:1) to receive or not receive self-paced pattern recognition training in skin cancer diagnostics using an LIIR with patient cases through a quiz-based smartphone app during an 8-day period. The participants’ ability to diagnose skin cancer was evaluated using a 12-item multiple-choice questionnaire prior to and 8 days after the educational intervention period. Their thoughts on the use of dermoscopy were assessed using a study-specific questionnaire. A learning curve was calculated through the analysis of data from the mobile app.

**Results:**

On average, participants in the intervention group spent 2 hours 26 minutes quizzing digital patient cases and 41 minutes reading the educational material. They had an average preintervention multiple choice questionnaire score of 52.0% of correct answers, which increased to 66.4% on the postintervention test; a statistically significant improvement of 14.3 percentage points (*P*<.001; 95% CI 9.8-18.9) with intention-to-treat analysis. Analysis of participants who received the intervention as per protocol (500 patient cases in 8 days) showed an average increase of 16.7 percentage points (*P*<.001; 95% CI 11.3-22.0) from 53.9% to 70.5%. Their overall ability to correctly recognize malignant lesions in the LIIR patient cases improved over the intervention period by 6.6 percentage points from 67.1% (95% CI 65.2-69.3) to 73.7% (95% CI 72.5-75.0) and their ability to set the correct diagnosis improved by 10.5 percentage points from 42.5% (95% CI 40.2%-44.8%) to 53.0% (95% CI 51.3-54.9). The diagnostic confidence of participants in the intervention group increased on a scale from 1 to 4 by 32.9% from 1.6 to 2.1 (*P*<.001). Participants in the control group did not increase their postintervention score or their diagnostic confidence during the same period.

**Conclusions:**

Self-paced pattern recognition training in skin cancer diagnostics through the use of a digital LIIR with patient cases delivered by a quiz-based mobile app improves the diagnostic accuracy of PCPs.

**Trial Registration:**

ClinicalTrials.gov NCT05661370; https://classic.clinicaltrials.gov/ct2/show/NCT05661370

## Introduction

Skin cancer diagnostics and skin tumor triage are challenging, and mastery often requires many years of clinical practice. A previous study by our research group has shown that it takes 6-10 years to become proficient [1], while others have not found any correlation between primary care physicians’ (PCPs) ability to diagnose and manage patients with skin cancer and years of clinical experience [2], rendering “bedside” education insufficient at best. Several strategies to help PCPs with this challenging task have been developed, including mnemonic techniques, checklists [3-10], and diagnostic Artificial Intelligence [11,12], neither of which can stand alone [13,14]. If properly trained, inspection of skin lesions using dermoscopy is associated with a higher detection rate of melanoma [15-17], a reduction in needed referrals and excisions [18-20], better management of pigmented skin lesions [21,22], and this provides an increase in melanoma sensitivity without a decrease in specificity [23] that is cost-effective [24]. Furthermore, comparing lesions over time using digital dermoscopy offers earlier detection [25]. Yet despite the strong evidence many PCPs still do not use dermoscopy [22,26], few have received training in dermoscopy [22], and those that use a dermoscope often do so without training [27], which has been suggested to decrease diagnostic ability [28]. Training in dermoscopy was the focus of a recent Cochrane review which highlights the need for research to identify the optimal approach [15]. Courses teaching dermoscopy with physical attendance improve PCPs’ skills in skin cancer diagnostics in the short term but require refresher training to maintain the acquired skills [29]. Many different types of web-based or electronic learning have been used to improve the diagnostic abilities of PCPs [30]. A trial by our research group has recently shown that medical students with no clinical experience, by spending approximately 3.5 hours with a newly developed educational mobile app that presents the user with a digital large-scale interactive image repository (LIIR) containing patient cases and educational material, improved their diagnostic accuracy significantly from 31% to 52% [31]. This has never been tested among PCPs. With this study, we aimed to examine if self-paced training in skin cancer diagnostics using a LIIR with patient cases improves PCPs’ diagnostic skills. In addition, we will investigate their ability to set the correct diagnosis (diagnostic accuracy) and to correctly classify digital patient cases as benign or malignant, their change in diagnostic confidence, and measure their time spent on the intervention.

## Methods

### Study Design and Setting

This randomized controlled trial used block randomization in blocks of 4, using a web-based randomizer [32] to generate a random number sequence of numbers 1 through 4. Participants were allocated continuously and sequentially as they signed up to either “Intervention” or “Control” with a 3:1 ratio, as we anticipated a large effect size of our primary outcome and desired further power for the analysis of data from the intervention group. Study recruitment was open for 30 days from mid-November to mid-December 2021.

### Participant Recruitment and Intervention

Eligible PCPs (doctors currently working in the primary care sector) were recruited at a conference in November 2021 (Lægedage) in Denmark by the speakers at 3 skin cancer and melanoma sessions. Interested physicians scanned a QR code and signed up to receive information material and an invitation to participate in the study by email, including a link to a web-based survey (Google Forms, Google Ireland Limited, 2022) which contained a consent form. The survey also included questions about their experience with and use of dermoscopy, including their confidence in their diagnostic abilities on a scale from 1 (low) to 4 (high), and ended with a skin cancer multiple choice questionnaire (MCQ) including 12 patient cases from a list of 25 skin lesion cases with previously established validity evidence [1]. The maximum number of points acquired on the test is 12, indicating high diagnostic accuracy.

Participants in the intervention group were invited to one of several web-based initiation meetings where they were instructed on how to download, install, sign up, and access the LIIR through a quiz-based smartphone app for practicing skin cancer diagnostics. Participants could also download and install the app independently using a pdf-guide. After installation, the participants in the intervention group were given 8 days of access from the day of their sign-up in the app, in which they were asked to diagnose 500 digital patient cases. They were sent email reminders on days 4, 7, and 8. After the 8 days, they were told to abstain from using the app for 8 days (washout period) before answering a final skin cancer MCQ with 12 new cases.

**Figure 1:**
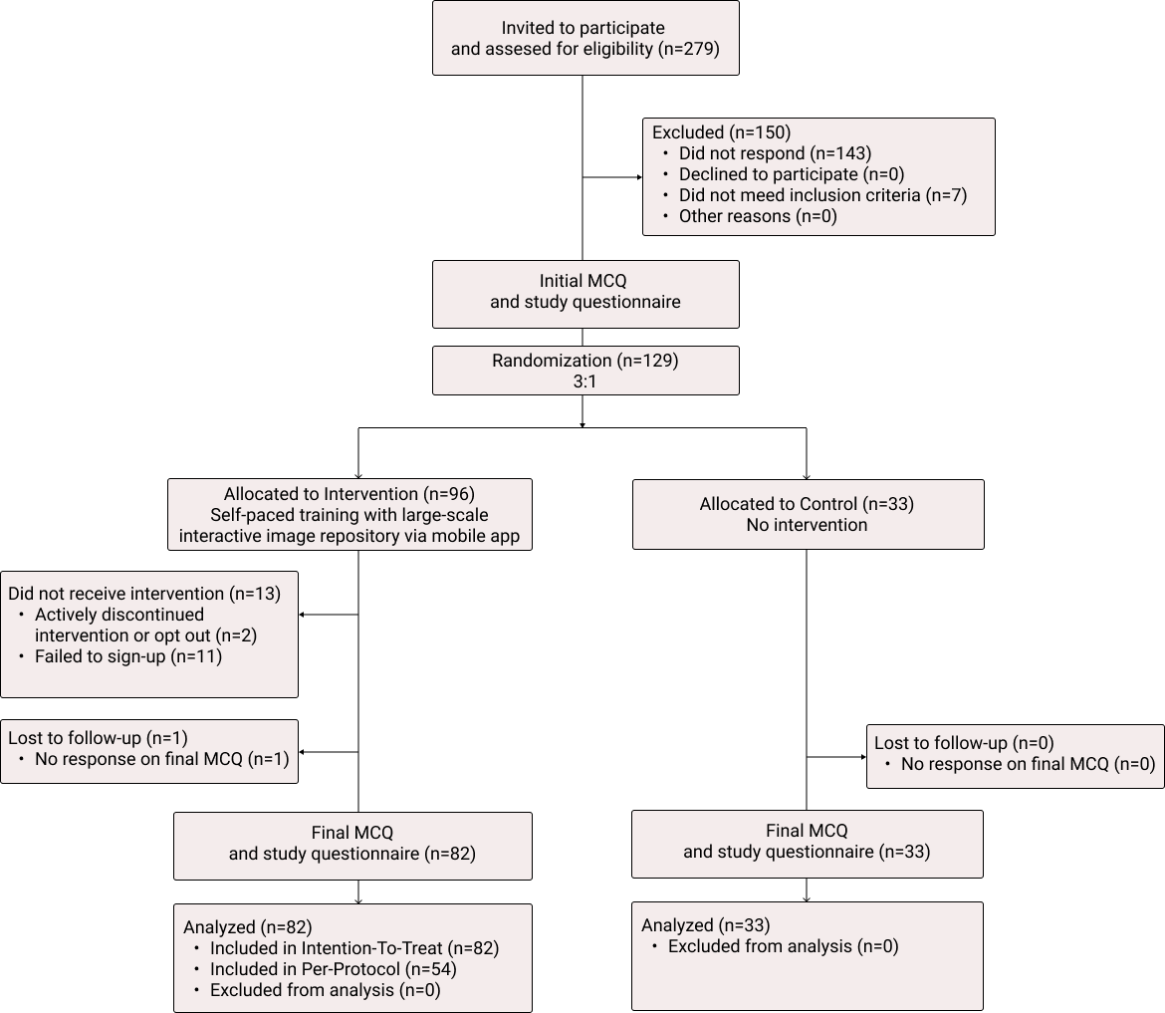
Consort Diagram. Of the 96 participants allocated to receive the intervention, 13 did not receive it at all and did not finish the final MCQ, one did not finish the final MCQ, and 28 only received the intervention partially, resulting in 82 participants included in the intention-to-treat analysis and 54 in the per-protocol analysis. MCQ: multiple choice questionnaire.

Participants in the control group continued their clinical practice as usual. They were not given access to the LIIR nor received any intervention before they completed the final skin cancer MCQ 16 days after their initial MCQ. See the study diagram in Figure 1.

### Outcomes

The trial’s primary outcome was the participants’ score on a skin cancer MCQ before and after the intervention, with both per-protocol and intention-to-treat analysis.

Secondary outcomes included the progression of the participants’ ability to correctly diagnose and classify digital patient cases in the LIIR across the intervention period, descriptive analysis of which diagnoses were most commonly misclassified and misdiagnosed, change in the participants’ diagnostic confidence, and the average time spent training.

### Blinding

Participants were unaware of their allocation until they had answered the initial questionnaire and MCQ. No measurements were taken to blind the principal investigator when receiving questionnaire responses and MCQ test results or when performing the statistical analysis comparing the 2 groups.

### Large-Scale Interactive Image Repository

For the educational intervention in this study, we used the educational mobile app Dermloop Learn (Melatech ApS) [33] and its LIIR that has 3 main functionalities: Quizzes with anonymized digital patient cases, written learning modules, and user tracking.

**Figure 2:**
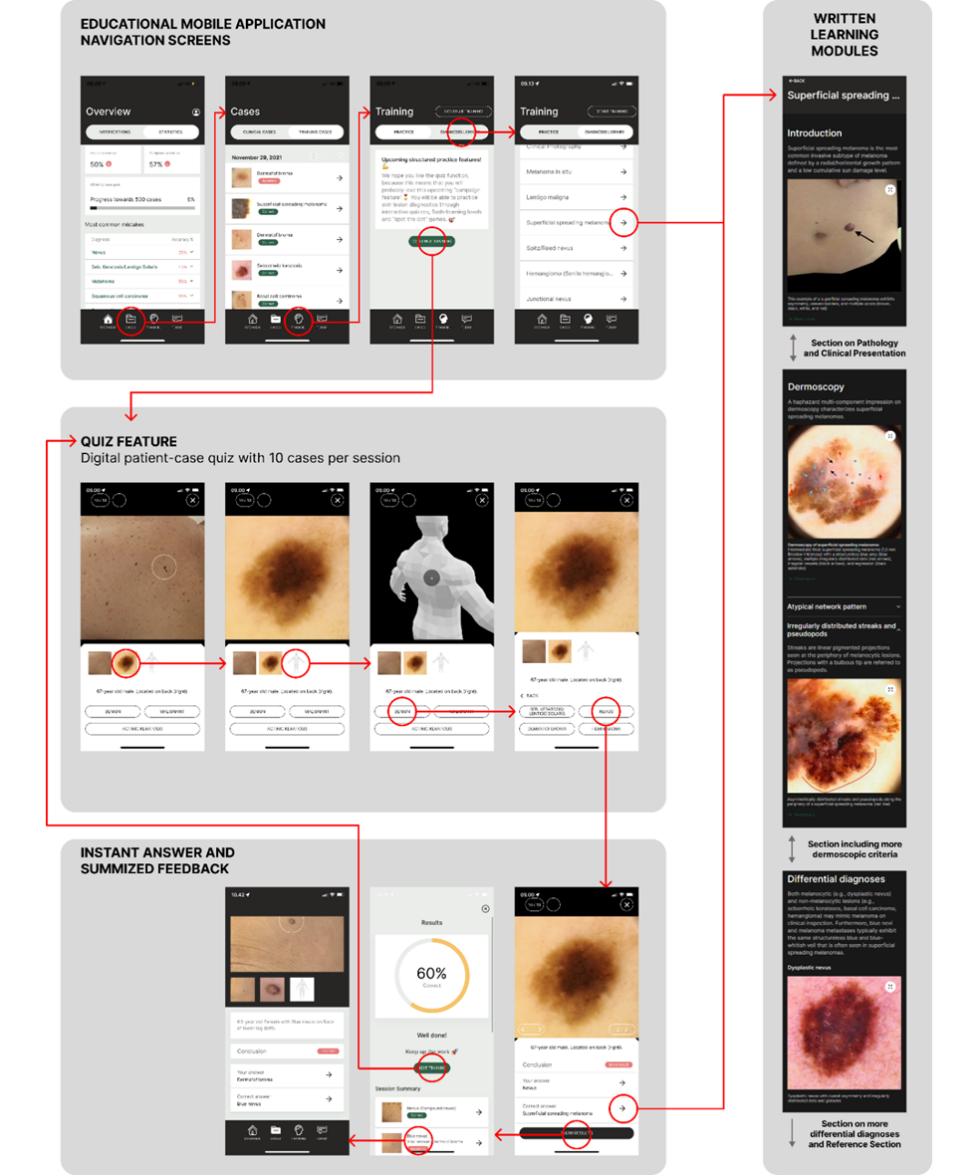
The quiz-based smartphone app uses a digital large-scale interactive image repository, Dermloop Learn, in the version used in the study. The red circles indicate where a user would “press” to progress from one screen to another, and the red arrows indicate which screen is shown next. On the “Overview” screen, the user can navigate to “Cases” or “Training” to either see a list of previously encountered cases or start or continue a training session or access the “Diagnosis Library,” which contains a list of the 36 written learning modules on the included diagnoses and subdiagnoses. When using the “Quiz Feature,” the user starts a session with 10 patient cases. For each case, a clinical and dermoscopic image and the location of the lesion on a 3D avatar are shown. When the user presses benign or malignant, an array of new buttons representing the included benign or malignant differential diagnoses appear. When the user presses a diagnosis, they receive immediate feedback and buttons to the written learning modules on both the chosen and correct diagnosis. Once all 10 cases in a session are diagnosed a short report including the user’s diagnostic accuracy for the session and a list of the cases is presented. From here, the user can reexamine their answered cases or start a new session.

The app used a library of 2376 digital patient cases with a diagnosis (either confirmed by histopathology or clinical consensus of 2 or more clinicians) belonging to 1 of 7 diagnosis groups (nevus, seborrheic keratosis (SK) or solar lentigo, dermatofibroma, hemangioma, melanoma, basal cell carcinoma or squamous cell carcinoma) where any subtype was considered correctly diagnosed if the participant answered the diagnosis-group correctly (eg, “Melanoma” was correct for both superficial spreading melanoma and lentigo maligna). Each digital patient case included the age and gender of the patient, a clinical and a dermoscopic image of the lesion, and its location on a 3D avatar; all of which are referred to as a “case.” See Figure 2 for an overview and example.

The participants trained in pattern recognition (quizzing) with sessions containing 10 cases selected from the library. One case was presented at a time with a clinical image, dermoscopic image, and the lesion’s location. The participant selected benign (and picked the suspected diagnosis from among nevus, SK or solar lentigo, dermatofibroma, or hemangioma) or malignant (and picked the suspected diagnosis from among melanoma, basal cell carcinoma, or squamous cell carcinoma). Immediately after answering a case, the user got feedback on whether the answer was correct or not, what the correct answer was, and an option of being taken to a written learning module on the correct and incorrect answer, respectively. The smartphone app has written learning modules on 36 diagnoses and subdiagnoses corresponding to the diagnoses of the cases contained in the LIIR. Each written learning module has an introductory section followed by sections on histopathology, clinical presentation, dermoscopic features, and differential diagnoses to the diagnosis, all including illustrations or examples from the LIIR.

As the user answered cases, the app compiled a list of the diagnoses for which the individual user had the most difficulty giving correct answers. The list was shown on the front page of the app (Overview page in Figure 2), nudging the user towards reading the corresponding written learning modules. The app also tracked how many cases each user had diagnosed, their answer to each case, how long they spent with each case, what written modules they opened, and how long they spent reading each time they opened a written module.

The Dermloop Lean app underwent no changes, updates, or bug fixes and there was no downtime during the trial period.

### Statistical Analysis

Sample size and power calculations were done by assuming a 50% diagnostic accuracy at baseline. Based on the preliminary results of a previous study using the same intervention on medical students [31], we anticipated a 20% (SD 15%) effect of the educational intervention, which should let us show a difference between the groups by including 96 participants with a 3:1 allocation ratio with a statistical significance level of .05 and 80% power.

Based on the methods of the similar and recent study by our research group [31] using Generalized Estimating Equations, learning curves of the participants’ probability to diagnose cases correctly were expressed as linear splines with a single knot at 100 cases using a logistic regression model with random intercept with correct diagnosis or not as an outcome. A similar model was made with the correct classification of each case as malignant or benign as the outcome. The participants’ time spent reading and quizzing was summed separately for each 100-case period.

MCQ scores for both groups before and after the study period were compared using 2 tailed Welch 2 Sample *t* test.

In this study, the intention-to-treat analysis included those individuals who participated and also answered the final questionnaire. The per-protocol analysis included those individuals who completed the stipulated 500 cases.

Statistical analyses were performed in R [34] (version 4.2.0; R Foundation for Statistical Computing) using GEE R Pack [35,36] for the primary analysis and learning curves and Excel (Microsoft Corp) [37] for descriptive statistics of study questionnaire responses.

### Ethical Considerations

The study was conducted in accordance with the principles of the Declaration of Helsinki. All participating doctors were given oral and written information about the study. All participants were informed that filling out the survey was seen as consent to participate. There was no financial compensation for the participants in the study. The study intervention is an educational intervention for medical doctors with no patient participation which does not require ethics board review [38].

## Results

### Participant Demographics and Study Flow

A total of 279 people applied for more information about the study, 135 accepted the invitation and finished the initial questionnaire, of which 7 were excluded as they were not doctors from the primary care sector. Initially, 96 were randomized to the intervention and 33 to the control group. From the intervention group, 84 subsequently set up a user profile granting them access to the smartphone app and its LIIR, of which 82 answered the final MCQ and were included in the intention-to-treat analysis (see CONSORT [Consolidated Standards of Reporting Trials] diagram in Figure 1). Demographics on all participants and their experience with dermoscopy can be found in Tables 1 and 2. The intervention group completed 467 cases on average (median 505, range 0-1679), and 54 participants completed 500 cases (as per protocol) or more and were included in the Per-Protocol analysis. All 33 participants from the control group finished the final MCQ. The time from the initial to the final MCQ was 21 and 19 days for the intervention and control groups, respectively.

**Table 1:** Participant demographics.

Participant demographics		Intervention (n=82)	Control (n=33)	*P* value^a^
Age (years), mean (range)		40.7 (27-73)	41.4 (26-63)	.69
**Gender, n (%)**				
	Male	36 (44)	17 (52)	.59
	Female	46 (56)	16 (48)	.59
**Clinical position, n (%)**				
	Intern (KBU)^b^	9 (11)	4 (12)	>.99
	Resident (Intro)^c^	1 (1)	5 (15)	.01
	Specialist Registrar^d^	29 (35)	4 (12)	.02
	Consultant	43 (52)	20 (61)	.56

^a^*P* value derived from a chi-square or Fisher exact test.

^b^Klinisk Basisuddannelse,” the first year after graduating.

^c^Introduction employment position, the second year after graduating.

^d^Typically the third-seventh year after graduating.

**Table 2:** Participants’ responses on the questionnaire regarding experience with skin cancer diagnostics including use of and thoughts about dermoscopy.

Questionnaire on skin cancer diagnostics and use of dermoscopy		Intervention (n=82)	Control (n=33)	*P* value^a^
Years of experience with skin diagnostics, mean (range)		5.7 (0-30)	6.6 (0-20)	.48
**Experience with dermoscopy, n (%)**				
	0-3 months	26 (32)	11 (33)	>.99
	4-11 months	17 (21)	8 (24)	.87
	1-2 years	21 (26)	5 (15)	.33
	3-5 years	15 (18)	7 (21)	.92
	6-10 years	3 (4)	2 (6)	.62
**Training in skin cancer diagnostics or use of dermoscopy, n (%)**				
	None	15 (18)	6 (18)	>.99
	Peer-To-Peer training	40 (49)	16 (48)	>.99
	Self-initiated learning	51 (62)	22 (67)	.81
	Web-based course in dermoscopy	2 (2)	3 (9)	.14
	Physically attended a course in tumors of the skin	33 (40)	12 (36)	.86
	Physically attended a course in dermoscopy	9 (11)	4 (12)	>.99
**Patients per week seen on suspicion of skin cancer, n (%)**				
	0-2	50 (61)	16 (48)	.31
	3-4	26 (32)	13 (39)	.57
	5-6	4 (5)	4 (12)	.22
	>6	0 (0)	0 (0)	>.99
**Patients referred to a dermatologist or plastic surgeon each week on suspicion of skin cancer, n (%)**				
	0-2	78 (95)	32 (97)	>.99
	3-4	4 (5)	1 (3)	>.99
	5-6	0 (0)	0 (0)	>.99
	>6	0 (0)	0 (0)	>.99
**Access to some form of teledermatology, n (%)**				
	No	25 (30)	14 (42)	.31
	Yes, for a select number of dermatological issues	13 (16)	4 (12)	.77
	Yes, for nonpigmented skin lesions	20 (24)	6 (18)	.64
	Yes, for all skin conditions	23 (28)	8 (24)	.85
**Preferred technique or algorithm when inspecting potential malignant melanoma lesions, n (%)**				
	ABCDE^b^	71 (87)	25 (76)	.26
	Ugly Duckling^c^	44 (54)	19 (58)	.86
	Dermoscopic pattern recognition^d^	19 (23)	11 (33)	.37
	No preferred technique	5 (6)	2 (6)	>.99
**Preferred type of inspection when evaluating pigmented skin lesions, n (%)**				
	Dermoscopic inspection	60 (73)	26 (79)	.70
	Naked eye inspection	40 (27)	74 (21)	.70
**Preferred type of inspection when evaluating nonpigmented skin lesions, n (%)**				
	Dermoscopic inspection	48 (59)	22 (67)	.55
	Naked eye inspection	52 (41)	78 (33)	.55
**Access to dermoscope, n (%)**				
	No	9 (11)	6 (18)	.36
	Shared dermoscope in the clinic	42 (51)	12 (36)	.22
	A colleague has one that I with difficulty can burrow	1 (1)	0 (0)	>.99
	A colleague has one that can burrow easily	13 (16)	5 (15)	>.99
	In my consultation room	17 (21)	10 (30)	.39
Diagnostic confidence on a scale from 1 (low) to 4 (high)		(1.6)	(1.6)	.81
**Main advantage of using dermoscope, n (%)**				
	Fewer referrals to the dermatologist	17 (21)	6 (18)	.96
	Earlier recognition of skin and mole cancer	43 (52)	18 (55)	>.99
	Use of the dermoscope puts me at ease	13 (16)	4 (12)	.77
	Use of the dermoscope puts the patient at ease	4 (5)	1 (3)	>.99
	No advantages	1 (1)	1 (3)	.49
**Main disadvantage of using dermoscope, n (%)**				
	Using a dermoscope requires experience	53 (65)	15 (45)	.09
	The dermoscope is expensive	3 (4)	6 (18)	.02
	The dermoscope is technically challenging to use	6 (7)	1 (3)	.67
	Using the dermoscope is time-consuming	0 (0)	1 (3)	.29
	No disadvantages	15 (18)	8 (24)	.64

^a^*P* value derived from a chi-square or Fisher exact test.

^b^"ABCDE” is the acronym for Asymmetry, Boarder, Color, Diameter, and Evolution or Elevation, a commonly used acronym in diagnosing melanoma without the use of a dermoscope.

^c^"Ugly Duckling” is a technique widely used to evaluate if a lesion is suspicious from a patient’s other nevi.

^d^"Dermoscopic Pattern Recognition” refers to any technique used by the respondent to recognize dermoscopic features (patterns) indicating malignancy.

### Intervention Effect

**Figure 3:**
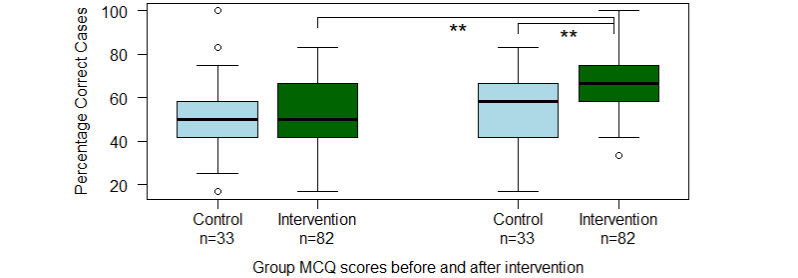
Box-and-whisker plot showing median scores (thick black horizontal lines), 95% CI (boxes), range (vertical whiskers), and outliers (circle) for the participants’ MCQ scores before and after the intervention. Asterix indicates a statistically significant difference on 2-tailed Welch t test. MCQ: multiple choice questionnaire.

The average MCQ score of the PCPs in the intervention group (intention-to-treat analysis) improved from 52.0% (6.2 correct answers out of 12) to 66.4% (8.0 correct answers out of 12); an improvement of 14.3 percentage points (95% CI 9.8-18.9; *P*<.001). Those participants who diagnosed 500 patient cases or more (per-protocol analysis) on average improved from 53.9% (6.5 correct answers out of 12) to 70.5% (8.5 correct answers); an improvement of 16.6 percentage points (95% CI 11.3-22.0; *P*<.001). The MCQ score of the control group did not improve during the study period (*P*=.94). See Figure 3 for a box-and-whiskers plot of each group’s initial and final MCQ percentages.

**Figure 4:**
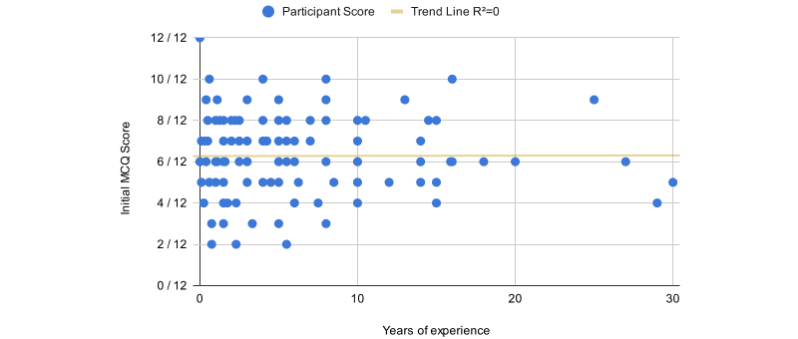
Scatter plot of all participants’ scores on initial MCQ and their years of experience diagnosing skin and mole cancer. The trend line reveals no correlation between the 2. MCQ: multiple choice questionnaire.

A post hoc analysis of the participant’s initial MCQ score and their years of experience diagnosing skin lesions found no correlation, as shown in Figure 4.

**Figure 5:**
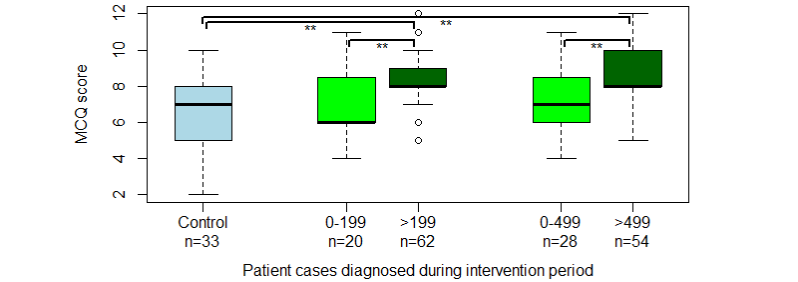
Box-and-whisker plot of participants’ MCQ scores separated by their number of diagnosed patient cases. The figure shows median scores (thick black horizontal lines), 95% CI (boxes), range (vertical whiskers), and outliers (circle) for the participants’ scores on their final MCQ test. Asterix indicates a statistically significant difference on 2 tailed Welch t test. MCQ: multiple choice questionnaire.

Analysis revealed a statistically significant difference in MCQ scores between participants who diagnosed less than the protocolled 500 cases and participants who diagnosed 500 or more (1.5 points, *P*<.001; 95% CI 0.7-2.3), and post hoc analysis found similar results between participants who diagnosed less or more than 200 cases (1.5 points, *P*=.004; 95% CI 0.5-2.4), respectively, as depicted in Figure 5. There was no statistically significant difference between the MCQ scores of those participants that diagnosed between 449-549 cases and those that did 550 or more (*P*=.12).

**Figure 6:**
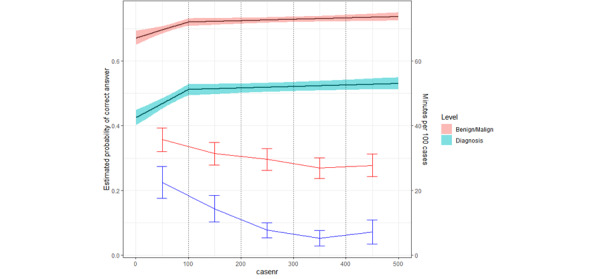
Learning curves for participants in the intervention group. The figure depicts the users’ progressive means (solid black lines) and 95% CI (teal and light red areas). The solid red and solid dark blue lines, respectively, depict time spent quizzing patient cases and time spent reading written learning modules separated into 100-case segments with 95% CI shown using whiskers.

The participants in the intervention group performed a total of 39,022 diagnostic evaluations during the study period. When comparing the participants’ answers on the first (case 0-50) and last (case 451-500) 50 cases, we found that their probability of correctly classifying a case as benign or malignant increased by 6.6 percentage points from 67.1% (95% CI 65.0%-69.3%) to 73.7% (95% CI 72.5%-75.0%). Their probability of setting the correct diagnosis (nevus, melanoma, dermatofibroma, etc) increased by 10.5 percentage points from 42.5% (95% CI 40.2%-44.8%) to 53.0% (95% CI 51.3%-54.9%). Learning curves were most steep during the first 100 cases, as shown in Figure 6.

A descriptive analysis of the 39,022 diagnostic evaluations made by the participants during the entire training period showed that 72.6% of the malignant cases were correctly classified as malignant. Lesions were misclassified as malignant or benign 26.9% and 27.4% of the time, respectively. The most often misclassified benign lesions were seborrheic keratoses, compound nevi, and junctional nevi, which were classified as malignant in 31.9%, 28.9%, and 27.8% of the assessments, respectively. Lentigo maligna, nodular melanoma, and melanoma in situ were misclassified as benign in 48.7%, 42.2%, and 42.1% of the assessments, respectively. Participants guessed a different diagnosis than the correct diagnosis most commonly when assessing dermal nevi (misdiagnosed in 66.1% of cases), nodular melanoma (misdiagnosed in 63.4% of cases), and lentigo maligna (misdiagnosed in 61% of cases). See Table 3 for further details on the distribution of the participants’ answers on these and all other diagnoses.

**Table 3:** The participants’ 39,022 case answers were distributed across the true diagnoses with percentage, number of encounters, and totals for each row. Explanatory example: Of the 6194 times a seborrheic keratosis was seen, they were answered correctly as a seborrheic keratosis 50% of the time (3087 instances), and correctly classified as benign 68% of the time (4218 instances) and misclassified as malignant 32% of the time (1976 instances).

True diagnosis			Distribution of participants’ answers																				
			Benign, n (%)											Malignant, n (%)									Grand total, n (%)
			Dermatofibroma		Hemangioma		Nevus		SK^a^ or Lentigo solaris		Benign total		BCC^b^			Melanoma		SCC^c^		Malignant total			
**Benign**																							
	Dermatofibroma	*1998 (51)* ^d^		102 (3)		480 (12)		400 (10)		2980 (76)		332 (8)			438 (11)		176 (4)		946 (24)		3926 (100)		
	Hemangioma	234 (6)		*2539 (66)*		224 (6)		64 (2)		3061 (79)		280 (7)			280 (7)		239 (6)		799 (21)		3860 (100)		
	Blue nevus	45 (7)		53 (8)		*350 (52)*		48 (7)		496 (74)		24 (4)			150 (22)		2 (0)		176 (26)		672 (100)		
	Compound nevus	300 (6)		55 (1)		*2593 (49)*		846 (16)		3794 (71)		125 (2)			1384 (26)		34 (1)		1543 (29)		5337 (100)		
	Dermal nevus	58 (12)		41 (8)		*167 (34)*		130 (26)		396 (80)		44 (9)			39 (8)		14 (3)		97 (20)		493 (100)		
	Junctional nevus	30 (3)		5 (0)		*661 (57)*		141 (12)		837 (72)		9 (1)			309 (27)		4 (0)		322 (28)		1159 (100)		
	Spitz nevus	35 (19)		12 (6)		*84 (45)*		6 (3)		137 (74)		4 (2)			45 (24)		0 (0)		49 (26)		186 (100)		
	Lentigo solaris	79 (5)		0 (0)		279 (18)		*836 (53)*		1194 (75)		80 (5)			279 (18)		36 (2)		395 (25)		1589 (100)		
	SK	337 (5)		207 (3)		587 (9)		*3087 (50)*		4218 (68)		536 (9)			949 (15)		491 (8)		1976 (32)		6194 (100)		
	Benign total	3116 (13)		3014 (13)		5425 (23)		5558 (24)		*17,113 (73)*		1434 (6)			3873 (17)		996 (4)		6303 (27)		23,416 (100)		
**Malignant**																							
	BCC	332 (8)		178 (4)		122 (3)		453 (11)		1085 (27)		*1597 (40)*			440 (11)		877 (22)		2914 (73)		3999 (100)		
	Lentigo maligna	3 (1)		2 (1)		21 (8)		104 (39)		130 (49)		26 (10)			*103 (39)*		8 (3)		137 (51)		267 (100)		
	LMM^e^	2 (2)		0 (0)		15 (11)		30 (23)		47 (36)		8 (6)			*74 (56)*		3 (2)		85 (64)		132 (100)		
	Melanoma in situ	47 (2)		4 (0)		494 (26)		267 (14)		812 (42)		57 (3)			*1040 (54)*		22 (1)		1119 (58)		1931 (100)		
	Nod. melanoma	1 (0)		63 (21)		52 (17)		13 (4)		129 (42)		34 (11)			*112 (37)*		31 (10)		177 (58)		306 (100)		
	SSM^f^	187 (4)		100 (2)		601 (12)		623 (12)		1511 (30)		284 (6)			*3132 (62)*		144 (3)		3560 (70)		5071 (100)		
	SCC	248 (6)		100 (3)		20 (1)		198 (5)		566 (15)		1069 (27)			110 (3)		*2155 (55)*		3334 (85)		3900 (100)		
	Malignant total	820 (5)		447 (3)		1325 (8)		1688 (11)		4280 (27)		3075 (20)			5011 (32)		3240 (21)		*11,326 (73)*		15,606 (100)		
Grand total			3936 (10)		3461 (9)		6750 (17)		7246 (19)		21,393 (55)		4509 (12)			8884 (23)		4236 (11)		17,629 (45)		39,022 (100)	

^a^SK: seborrheic keratosis.

^b^BCC: basal cell carcinoma.

^c^SCC: squamous cell carcinoma.

^d^Italicized figures represent true positives.

^e^LMM: lentigo maligna melanoma.

^f^SSM: superficial spreading melanoma.

### Time Spent Quizzing and Reading

During the 8-day intervention period, participants from the intervention group, on average, spent 2 hours 26 minutes (ranging from 0 minutes to 8 hours 35 minutes) practicing pattern recognition (quizzing) and 41 minutes (ranging from 0 minutes to 3 hours 23 minutes) reading the written educational modules included in the app. The majority of the participants’ reading activity was done at the beginning of the period and fell drastically after the first 100 cases (solid blue line in Figure 4). The average time spent diagnosing each case decreased from 36 to 28 seconds (solid red line in Figure 4).

### Diagnostic Confidence

The participants in both groups initially had identical relatively low confidence in their diagnostic ability to diagnose skin lesions using a dermoscope of 1.6 on a scale from 1 (low) to 4 (high), which increased significantly for participants in the intervention group by 32.9% to 2.1% (*P*<.001) after the intervention, but not for the control group (*P*=.23).

## Discussion

### Principal Findings

Participants who used the LIIR increased their ability to set the correct diagnosis by 10.5 percentage points and their ability to correctly classify lesions as benign or malignant by 6.6 percentage points. The steepest part of the learning curve was seen during exposure to the first 100 patient cases. This is consistent with previous studies [31,39,40]. Our findings align with previous research, indicating no direct relationship between years of experience and diagnostic competence in assessing skin lesions [2].

This study of 115 PCPs is one of the largest randomized controlled trials testing an educational intervention on PCPs’ ability to diagnose skin cancer [30] and the most extensive study using self-paced learning with a LIIR as the main component in the educational intervention. The participants were given no compensation for their time spent, which was done primarily in their spare time. Despite the trial period extending across the busy time before and during Christmas and New Year, the study had a high level of adherence. Of the 96 participants randomized to the intervention, 82 (85%) received it. A total of 12 participants did not manage to download and install the mobile app, which was yet to be released to the general public at the time. Despite this, 54 (62%) received the educational intervention as per protocol, and there were no dropouts from the control group. This level of adherence is similar to or higher than that of previous studies with a comparable intervention [30,41-43]. Post hoc calculations revealed a 98.1% statistical power for the intention-to-treat and 99.8% for the per-protocol analysis.

### Limitations

Limitations of this study include the recruitment method of inviting doctors at continuing education sessions about skin cancer. These doctors were perhaps more interested in or concerned about skin cancer diagnostics than the general population of PCPs. Evidence of this was that our participants had a relatively high initial diagnostic accuracy, most (84%) reported having access to a dermoscope in their clinic, and the percentage of participants who had received training (not including peer-to-peer and self-initiated training) in dermoscopy was also relatively high (46%). The effect of the educational intervention would possibly be more distinct in the general PCP population, where a lack of specific training in skin cancer diagnostics is more common [22]. Our study did not test long-term retention, and it is likely that the acquired skills will fade without continued use of the educational material [29]. Another limitation was that the 2376 patient cases presented in the LIIR were extracted from a department of dermatology and therefore likely to be more difficult than what is generally seen by the PCP. Therefore, the diagnostic accuracy found in this study might not reflect the PCPs’ diagnostic accuracy on the patients they meet in their clinic. Another discrepancy between diagnosing cases using the LIIR and the clinical examinations of a patient’s skin is that when encountering an irregular nevus in the clinic, examination of the patient’s other nevi may reveal that the nevus resembles the patient’s other nevi and therefore not an “ugly duckling,” but rather “regularly irregular,” reducing the suspicion of melanoma. Yet, with these cases being evaluated 39,022 times, it does reveal the most common diagnostic pitfalls in the population and where to intensify future educational interventions: SK, early melanomas, and nodular melanoma.

It was our initial hypothesis that PCPs, who possess the ability to apply the educational material in a clinical context, would exhibit a steeper learning curve with a higher end point from using this educational tool compared to medical students [31]. Contrary to our hypothesis, we did not observe a difference in the learning curve between primary care providers and medical students. One potential reason for this could be that primary care providers have a greater awareness of the consequences of their diagnostic decisions, which may have limited their assessment of the patient cases. They may have reacted as in the clinical setting and rather chosen a more serious diagnosis than miss a potentially malignant lesion.

As PCPs are the first to triage patients with skin cancer, improving the diagnostic accuracy of PCPs is necessary as the incidence of skin cancer has been rising [44,45] and is expected to keep rising [46-48]. The underlying causes for this growing disease burden are likely multifactorial, including biological and demographic factors such as increased ultraviolet exposure and a rapidly aging population. Skin cancer screening and general awareness increase the number of patients referred and biopsied [49], straining an already hard-pressed health care system. Adding to that, it has been stipulated that revised histopathologic criteria and historic underdiagnosis [50] but also current overdiagnosis [49,51,52] may contribute significantly to the growing incidence of skin cancer. It has previously been shown that increased awareness of skin cancer of PCPs does not increase the number of patients biopsied or referred to the dermatologist, etc [53], but rather that education and use of a dermoscope reduce the number needed to biopsy to detect melanoma in the primary health care setting [54]. Our results show a marked increase in the participants’ diagnostic confidence, which often does not correlate with diagnostic competence. Yet, with PCPs feeling more confident in their abilities, they could engage in doing more digital sequential follow-up, which has higher diagnostic accuracy than single-appointment evaluations [25]. This could potentially reduce the number of referrals and biopsies even further, leading to fewer excisions and, thereby, fewer overdiagnosed “melanomas” [55,56].

With the results of this study, we can address one of the leading causes of reluctance toward using dermoscopy in general practice: The time needed for training [57]. As digital education is easily accessible, can be acquired flexibly and on-demand, and does not require the participant to travel to and from the educational institute, it might be a more efficient way of increasing the diagnostic accuracy of multiple participants over a short period at a low cost. Our results show that the time needed to improve diagnostic skills using dermoscopy might not be more than a few hours at one’s own pace.

The perspectives of these results are potentially quite important; however, this trial did not test whether the observed improvements in diagnostic accuracy transfer to the participants’ clinical diagnostic accuracy and, in turn, if it changed their clinical patient management. This transfer of knowledge and its effect on clinical patient management should be the focus of future research.

### Conclusions

Using self-paced training in skin cancer diagnostics using a digital LIIR with patient cases delivered by a quiz-based mobile app improves PCPs’ diagnostic skills and confidence. The time spent by each participant does not need to be very long, nor must it be done in 1 sitting. Significant improvements can be seen from an average of 3.5 hours over the course of 8 days.

## Appendix

Multimedia Appendix 1 CONSORT-EHEALTH checklist (V 1.6.1).

## Abbreviations

LIIR: large-scale interactive image repository
MCQ: multiple choice questionnaire
PCP: primary care physician
SK: seborrheic keratosis
